# Elevated circulating adiponectin levels do not prevent anxiety-like behavior in a PCOS-like mouse model

**DOI:** 10.1038/s41598-023-50503-8

**Published:** 2024-01-04

**Authors:** Manisha Samad, Joakim Ek, Stina Börchers, Jean-Philippe Krieger, Elisabet Stener-Victorin, Karolina P. Skibicka, Ingrid Wernstedt Asterholm, Anna Benrick

**Affiliations:** 1 Department of Physiology, Institute of Neuroscience and Physiology, Sahlgrenska Academy, University of Gothenburg, Box 423, 40530 Gothenburg, Sweden; 2 Institute of Veterinary Pharmacology and Toxicology, University of Zurich-VetSuisse, 8057 Zurich, Switzerland; 3 Department of Physiology and Pharmacology, Karolinska Institute, 17177 Stockholm, Sweden; 4 Department of Nutritional Sciences, Pennsylvania State University, University Park, PA USA; 5 Huck Institutes of the Life Sciences, Pennsylvania State University, University Park, PA USA; 6 School of Health Sciences, University of Skövde, 54128 Skövde, Sweden

**Keywords:** Physiology, Reproductive biology, Reproductive disorders, Endocrine reproductive disorders

## Abstract

Polycystic ovary syndrome (PCOS) is associated with symptoms of moderate to severe anxiety and depression. Hyperandrogenism is a key feature together with lower levels of the adipocyte hormone adiponectin. Androgen exposure leads to anxiety-like behavior in female offspring while adiponectin is reported to be anxiolytic. Here we test the hypothesis that elevated adiponectin levels protect against the development of androgen-induced anxiety-like behavior. Pregnant mice overexpressing adiponectin (APNtg) and wildtypes were injected with vehicle or dihydrotestosterone to induce prenatal androgenization (PNA) in the offspring. Metabolic profiling and behavioral tests were performed in 4-month-old female offspring. PNA offspring spent more time in the closed arms of the elevated plus maze, indicating anxiety-like behavior. Intriguingly, neither maternal nor offspring adiponectin overexpression prevented an anxiety-like behavior in PNA-exposed offspring. However, adiponectin overexpression in dams had metabolic imprinting effects, shown as lower fat mass and glucose levels in their offspring. While serum adiponectin levels were elevated in APNtg mice, cerebrospinal fluid levels were similar between genotypes. Adiponectin overexpression improved metabolic functions but did not elicit anxiolytic effects in PNA-exposed offspring. These observations might be attributed to increased circulating but unchanged cerebrospinal fluid adiponectin levels in APNtg mice. Thus, increased adiponectin levels in the brain are likely needed to stimulate anxiolytic effects.

## Introduction

Polycystic ovary syndrome (PCOS) has traditionally been linked to reproductive and metabolic disorders, including obesity^1^. More recently, the association between PCOS and mental health issues has gained attention^2,3^. Women with PCOS often experience a lower health-related quality of life and increased scores of depressive and anxiety symptoms^4,5^. The lifetime prevalence of anxiety and depression is significantly elevated in women with PCOS (up to 76–80%) compared to women without PCOS, and this increase is also significant after correction for body mass index^5,6^.

Hyperandrogenism is a key feature of PCOS^1^, and evidence from both clinical and preclinical studies indicates a causative role for androgen excess in the development of PCOS. The hormonal imbalance persists during pregnancy, potentially affecting fetal development and increasing the risk of mood disorders^7^ and metabolic dysfunction in the offspring later in life. Intriguingly, our research has demonstrated that daughters of PCOS-affected women have an increased likelihood of developing PCOS^8^, and the offspring of women with PCOS face an elevated risk of being diagnosed with neuropsychiatric disorders such as attention-deficit/hyperactivity disorder (ADHD), autism spectrum disorders, and tic disorders compared with unrelated PCOS-unexposed offspring^7^, as well as anxiety and depression^9^. Interestingly, these associations with depression, ADHD and autism spectrum disorders were stronger in girls than boys^7,9^. These findings suggest that maternal androgen excess contributes to an increased risk for neuropsychiatric disorders in daughters of PCOS mothers.

Women with PCOS have reduced levels of adiponectin compared to women without PCOS^10^ and we have shown that low serum adiponectin levels are one of the strongest markers for insulin resistance in women with PCOS^11^. Accordingly, adiponectin overexpression prevents the development of metabolic disorders in PCOS-like mice^12^ and adiponectin appears to also have beneficial imprinting effects as prenatally androgenized (PNA) wild-type offspring from adiponectin-overexpressing dams exhibit enhanced metabolic functions^13^. However, the potential impact of adiponectin on behavior and mental health remains inadequately explored. Both androgens and adiponectin can pass the blood–brain barrier and influence anxiety and depression^14,15^. The interplay between elevated androgen levels, low adiponectin levels, and signs of depression and anxiety has been observed^6,10,16^, yet the precise mechanisms governing the impact of these hormones on mental health remain elusive. A comprehensive meta-analysis has revealed an inverse association between adiponectin levels and mental health, with low serum adiponectin associated with anxiety, mood, and trauma-related disorders^16^. Interestingly, the association with depressive disorders was stronger in females^16^.

The consistent emergence of a PCOS-like animal model following exposure to excessive prenatal androgens strongly supports the concept that hyperandrogenism plays a pivotal role in PCOS development. Anxiety-like behavior has been observed in PNA female offspring, with the mechanism likely involving hormone receptor expression changes in the amygdala^17,18^.

Therefore, a PNA mouse model serves as a platform to test the hypothesis that elevated adiponectin levels in female mice can protect against the development of androgen-induced anxiety-like behavior in the offspring.

## Results

### Study groups

**Figure 1:**
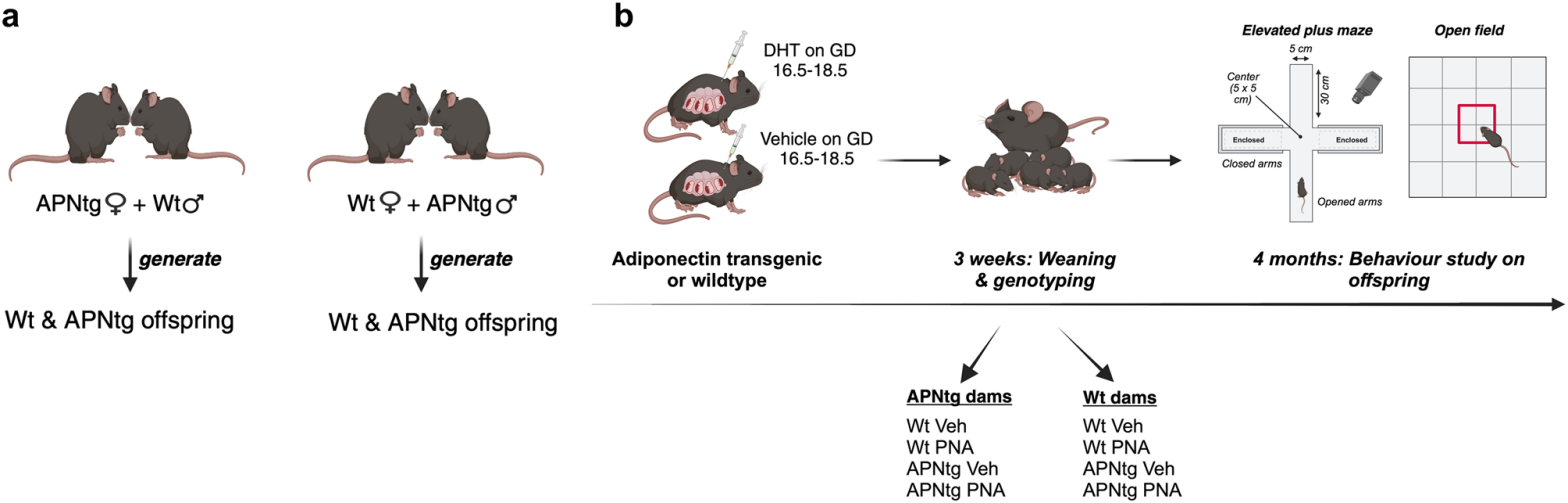
(**a**) Schematic representation of the breeding scheme generating wildtype (Wt) & adiponectin transgenic (APNtg) littermates. (**b**) Describes the overall study plan where the effects of adiponectin overexpression with and without prenatal androgenization (PNA) on anxiety-like behavior were studied using elevated plus maze and open field. Dihydrotestosterone; DHT (created with BioRender.com).

A transgenic mouse model overexpressing adiponectin (APNtg) was used to study the effect of adiponectin, with and without prenatal androgenization, on behavior. The presence of one APNtg allele is enough to overexpress adiponectin^19^, therefore APNtg and wt dams gave birth to both wt and APNtg offspring allowing us to study littermates (see breeding scheme and study outline in Fig. 1). The breeding resulted in 114 mice divided into 8 groups: wild type or APNtg offspring from wt dams exposed to vehicle injections (wt dams; wt Veh, n = 14, APNtg Veh, n = 10) or DHT-injections during gestation to induced PNA (wt dams; wt PNA, n = 26, APNtg PNA, n = 16), and wild-type or APNtg offspring from APNtg dams exposed to vehicle injections (APNtg dams; wt Veh, n = 18, APNtg Veh, n = 13) or DHT-injections to induce PNA (APNtg dams; wt PNA, n = 9, APNtg PNA, n = 8).

### Metabolic characteristics

**Figure 2:**
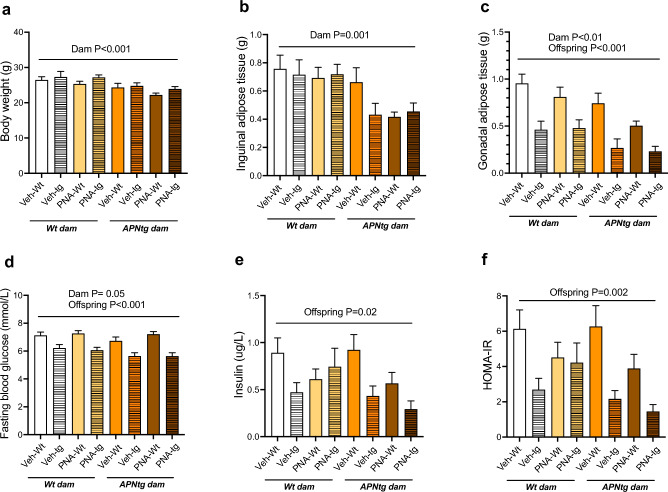
(**a**) Body weight, (**b**) inguinal adipose tissue mass, (**c**) gonadal adipose tissue mass, (**d**) fasting blood glusose, (**e**) insulin levels, and (**f**) homa-IR in 4-month-old wild-type (wt) and adiponectin transgenic (APNtg) female mice with and without prenatal androgenization (PNA). The main effect of PNA, the dams’ genotype and the offsprings’ genotype, as well as any interactions were measured using three-way ANOVA followed by Tukey post hoc test. Groups: wt or APNtg offspring from wt dams exposed to vehicle injections (wt-wt Veh, n = 14, wt-APNtg Veh, n = 10) or PNA (wt-wt PNA, n = 26, wt-APNtg PNA, n = 16), and wt or APNtg offspring from APNtg dams exposed to vehicle injections (APNtg-wt Veh, n = 18, APNtg-APNtg Veh, n = 13) or PNA (APNtg-wt PNA, n = 9, APNtg-APNtg PNA, n = 8).

There was a main effect of the dams’ genotype on body weight, fad pad weight, and fasting glucose levels in the offspring confirming previous findings that maternal adiponectin overexpression has imprinting effects on the offspring leading to improved metabolic functions^13^. The body weight (F_(1, 112)_ = 13.5, *P* < 0.001), subcutaneous inguinal white adipose tissue (F_(1, 105)_ = 11.5, *P* = 0.001) and visceral gonadal white adipose tissue (F_(1, 105)_ = 9.1, *P* = 0.003) weight, and blood glucose levels (F_(1, 104)_ = 3.9, *P* = 0.05) were lower in offspring from APNtg dams compared to offspring from wt dams (Fig. 2a–d).

The offsprings' genotype had a main effect on gonadal fat pad weight (F_(1, 105)_ = 24.2, *P* < 0.001), fasting glucose (F_(1, 104)_ = 42.4, *P* < 0.001) and insulin levels (F_(1, 102)_ = 5.3, *P* = 0.023) in the offspring at 4 months of age (Fig. 2c–e). Glucose and insulin levels were lower in APNtg mice, with a concurrent decrease in HOMA-IR (F_(1, 101)_ = 10.3, *P* = 0.002) (Fig. 2f), in line with adiponectins’ insulin-sensitizing effects^12^. There was a pronounced increase in BAT mass in APNtg mice compared to wildtypes (wt 58 ± 3 mg vs APNtg 144 ± 6 mg, *P* < 0.001, Supplemental Table S1), as previously described by us and others^20,21^. There was no difference in liver or ovary weight between the groups (Supplemental Table S1).

### Behavior

**Figure 3:**
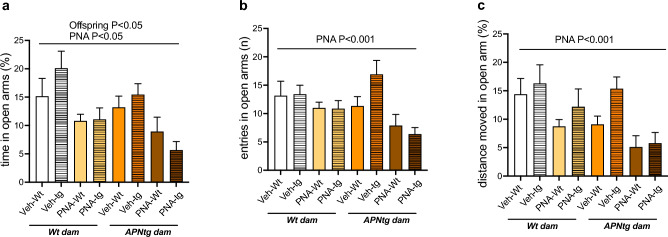
(**a**) Time spent in open arms (Dam *P* = 0.085), (**b**) number of entries into open arms, and (**c**) distance moved in open arms in 4-month-old wild-type (wt) and adiponectin transgenic (APNtg) female mice with and without prenatal androgenization (PNA). The main effect of PNA, the dams’ genotype and the offsprings’ genotype, as well as any interactions were measured using three-way ANOVA followed by Tukey post hoc test. Groups: wt or APNtg offspring from wt dams exposed to vehicle injections (wt-wt Veh, n = 14, wt-APNtg Veh, n = 10) or PNA (wt-wt PNA, n = 26, wt-APNtg PNA, n = 16), and wt or APNtg offspring from APNtg dams exposed to vehicle injections (APNtg-wt Veh, n = 18, APNtg-APNtg Veh, n = 13) or PNA (APNtg-wt PNA, n = 9, APNtg-APNtg PNA, n = 8).

There was a main effect of PNA on time spent in the open arms of the elevated plus maze (F_(1, 113)_ = 3.99, *P* = 0.048) where excess maternal androgens decreased the time the offspring spent in the open arms, indicating an anxiety-like behavior (Fig. 3a). There was a main effect of the offsprings’ genotype on time spent in the open arms of the elevated plus maze (F_(1, 113)_ = 4.53, *P* = 0.036). There was also a main effect of the dams’ genotype, that did not reach statistical significance, on time spent in the open arms of the elevated plus maze (F_(1, 113)_ = 3.03, *P* = 0.085) where elevated maternal adiponectin tended to decrease the time the offspring spent in the open arms (Fig. 3a). There was a main effect of PNA on the number of entries in the open arms (F_(1, 108)_ = 12.82, *P* < 0.001) where PNA decreased the number of entries (Fig. 3b). Moreover, there was a main effect of PNA (F_(1, 108)_ = 16.84, *P* < 0.001) on the distance moved in the open arms. PNA decreased the distance moved in the open arms (Fig. 3c). Thus, adiponectin overexpression in the offspring and/or the dam does not protect against androgen-induced anxiety-like behavior in the offspring.

There was no difference in the latency to enter, time spent in the center zone, or the number of entries into the center zone of the open field (Supplemental Table S2). There was a main effect on offsprings’ genotype on the distance moved in the center zone open field (*P* = 0.015) with interaction effects between dams’ genotype and PNA (F_(1, 110)_ = 6.40, *P* = 0.013) and offsprings’ genotype and PNA (F_(1, 110)_ = 6.77, *P* = 0.011). The total distance moved in the open field was similar between groups.

### Serum and CSF adiponectin levels

**Figure 4:**
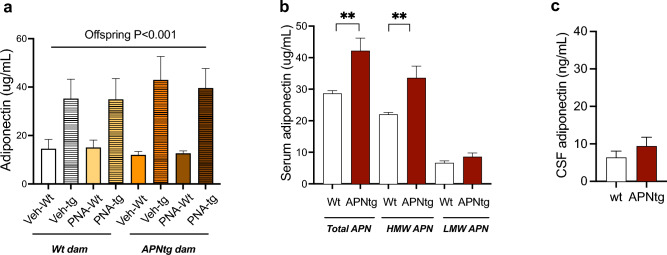
(**a**) Total serum adiponectin in 4-month-old wild-type (wt) and adiponectin transgenic (APNtg) female mice with and without prenatal androgenization (PNA), (**b**) high and low molecular weight adiponectin in serum, and (**c**) total adiponectin in CSF measured in wild-type (wt) and adiponectin transgenic (APNtg) female mice. The main effect of PNA, the dams’ genotype and the offsprings’ genotype on adiponectin levels, as well as any interactions, were measured using three-way ANOVA followed by Tukey post hoc test. Changes in serum and CSF adiponectin levels between wt and APNtg mice were measured by Student’s unpaired t-test, **P < 0.01.

Circulating adiponectin levels were two- to threefold higher in APNtg mice compared to wt (F_(1, 64)_ = 430.3, *P* < 0.001), with no effect of PNA exposure (Fig. 4a). There was an interaction between dams’ genotype and offsprings’ genotype (F_(1, 64)_ = 11.91, *P* < 0.001), where adiponectin levels were higher in APNtg offspring from APNtg dams. We then measured the fraction of high- (HMW) and low-molecular-weight (LMW) adiponectin and found that APNtg mice have more circulating HMW adiponectin but equal amounts of LMW adiponectin (Fig. 4b). However, the CSF adiponectin levels were similar between APNtg and wt mice (Fig. 4c), in line with previous findings that only low molecular forms of adiponectin can pass the blood–brain barrier^14,22^.

## Discussion

Emerging evidence suggests that PCOS may have its origins, at least in part, during fetal development^8^. Elevated maternal androgens are believed to play a pivotal role in this process, contributing to placental dysfunction and placing the developing fetus at an increased risk for lifelong psychiatric disorders^3,9^. During pregnancy, women with PCOS maintain high levels of circulating androgens, and these elevated levels are associated with changes in placental function, including reduced aromatase expression, which may lead to increased exposure of the fetus to testosterone^23,24^. Proper placental function is critical for fetal growth, and any alteration in its function can lead to abnormal fetal development and structural changes in the developing brain^18,25,26^, as well as other somatic tissues. Additionally, hyperandrogenism has been linked to altered white matter microstructure and compromised cognitive and neuropsychiatric function^26^. Offspring of women with PCOS have an increased risk of being diagnosed with ADHD, autism spectrum disorders, Tourette’s disorder, and chronic tic disorders compared with unrelated non-PCOS offspring^7,9,27–30^. These clinical observations strongly suggest that elevated maternal androgens affect the developing fetus and contribute to in utero programming of the developing brain.

In line with human research findings, excessive PNA exposure has consistently been shown to induce anxiety-like behavior in rodent offspring and to alter gene expression in their brains^9,17,18^. Since testosterone can be partly converted to estrogen, it may act on both androgen and estrogen receptors. In a study involving rats exposed to prenatal androgenization, simultaneous administration of tamoxifen, an estrogen receptor blocker, reversed the anxiety-like behavioral phenotype caused by PNA exposure^17^. This suggests that the effect is not solely mediated through androgen receptors and may involve estrogenic influences. To rule out an estrogenic effect, we utilized DHT, a nonaromatizable androgen.

Elevated levels of adiponectin in the dam impact placental function and metabolic processes, leading to metabolic imprinting effects, such as improved glucose regulation and decreased fat mass in the offspring (^13^ and this study). However, maternal overexpression of adiponectin did not prevent the development of androgen-induced anxiety-like behavior, suggesting that adiponectin does not provide protection against structural changes in the developing brain due to in utero androgenic exposure. If anything, offspring from dams overexpressing adiponectin exhibited slightly reduced mobility in the open arms of the EPM. In summary, while adiponectin influences placental function, its effects are unlikely to involve fetal androgen exposure or structural changes in the developing brain.

Our previous research, along with this study, demonstrates that increased fetal exposure to adiponectin programs offspring to have better metabolic health, resulting in notable effects on fat mass and glucose regulation^13^. The insulin-sensitizing action of adiponectin involves the activation of AMPK in the liver and increased β-oxidation in skeletal muscle^19^. Furthermore, APNtg mice exhibit enhanced clearance of circulating fatty acids and increased capacity of subcutaneous adipose tissue expansion when subjected to a high-calorie diet. These effects are mediated through PPARα signaling^21,31^. This enhanced metabolic flexibility, coupled with an increased capacity to store fat in subcutaneous adipose tissue prevents hepatic lipid accumulation and preserves insulin sensitivity in APNtg mice. Therefore, higher circulating levels of adiponectin protect against the metabolic disturbances associated with prenatal androgenization. Although previous research indicates that adiponectin has anxiolytic effects^32,33^, it is important to consider that behavioral changes must be mediated through central effects. While APNtg offspring did show a trend of spending more time in the open arms of the EPM, this behavior was only present in controls, not in mice exposed to excess androgens. There were no other behavioral effects associated with elevated adiponectin in the offspring. The presence of peripheral effects but a lack of central effects of adiponectin led us to investigate adiponectin levels in the CSF. Adiponectin levels in the CSF were approximately 0.1% of the serum concentration, a ratio similar to what has previously been described in humans and rats but lower than the 1% described in mice^14,22,34^. Despite significantly higher circulating adiponectin levels in APNtg mice, CSF levels remained similar to those in wild-type mice. There is controversy about whether or not adiponectin crosses the blood–brain barrier. A rise of CSF adiponectin was demonstrated after intravenous injection of adiponectin^34^ but this was later challenged when using sensitive radiotracer methods to quantify blood–brain barrier influx failed to detect significant permeation of adiponectin^35,36^. Adiponectin exists in three multimeric forms, with high-molecular-weight adiponectin being the most abundant and biologically active form^37^**,** while only the adiponectin low-molecular-weight hexamers are found in human CSF^22^. The presence of LMW adiponectin in human CSF confirms the ability of the LMW form to traverse the blood–brain barrier but whether it is through diffusion or active transport is yet to be determined. Moreover, the discovery that intracerebroventricular injection of LMW forms, as opposed to the collagenous tail fragment enabling HMW formation, exerts central effects in mice lends support to the notion that LMW adiponectin can cross the blood–brain barrier^34^. Since HMW adiponectin is too large to cross the blood–brain barrier, we measured both HMW and LMW isoforms of serum adiponectin. It became evident that the smaller low molecular forms of adiponectin, capable of crossing the blood–brain barrier, remained unaltered in APNtg mice. Contrary to our hypothesis, overexpression of adiponectin did not elicit anxiolytic effects in PNA-exposed offspring. This observation might be attributed to the absence of increased adiponectin levels in the CSF of APNtg mice, thereby hindering the establishment of a central anxiolytic effect. Another possible mechanism involves the expression of central adiponectin receptors. While it is established that adiponectin receptors are indeed present in the brain, some of adiponectin’s effects may also be mediated by receptors located outside nuclei impacting behavior or metabolism^32–34^. In mice, the expression of both adiponectin receptor-1 and receptor-2 are present in brain endothelial cells, and treatment with adiponectin reduces the secretion of interleukin-6 from these cells^36^. Thus, the anti-inflammatory properties of adiponectin offer an alternative explanation as to how systemic adiponectin could modulate blood–brain barrier function, thereby mediating central effects. It is important to note, however, that this explanation does not include anxiolytic effects in our model. To ascertain the potential anxiolytic properties of adiponectin, additional approaches such as utilizing a transgenic model that overexpress the LMW form of adiponectin, employing a brain-specific cre model to induce increased adiponectin levels in the brain, or administrating adiponectin intracerebroventricularly are necessary.

## Material and methods

### Animal model

Wild-type (wt) and adiponectin transgenic (APNtg) mice overexpressing adiponectin^19^ on a C57BL/6J background were maintained under standard housing conditions at the animal core facility at the University of Gothenburg. They had ad libitum access to food and water under controlled conditions (12-h light/dark cycle environment, and fixed temperature and humidity). All experiments were performed with permission from the Animal Ethics Committee of the University of Gothenburg and were performed according to the ARRIVE guidelines, the European Union guidelines for the care and use of laboratory animals (2010/63/EU), and the Swedish Board of Agriculture’s regulations and general advice of laboratory animals (L150).

### Study design

Females in the estrus phase, as determined by vaginal smears^38^, were mated overnight. Wt females were mated with APNtg males and APNtg females were mated with wt males. Pregnancy was confirmed by a distinct weight gain. On GD 16.5 the groups were subdivided and assigned to receive a subcutaneous injection in the interscapular area of vehicle or 250 μg 5α-Androstan-17β-ol-3-one (dihydrotestosterone (DHT), A8380, Sigma-Aldrich, St. Louis, USA) dissolved in 2.5 μl benzyl benzoate (B6630, Sigma-Aldrich) and 47.5 μl sesame oil (S3547, Sigma-Aldrich) for three days. Prenatal androgenization with DHT induces a PCOS-like phenotype in the offspring^18,39^. APNtg and wt dams gave birth to both wt and APNtg offspring. Female offspring were weaned at 4 weeks of age and genotyped as previously described^12^. The breeding scheme and study design are shown in Fig. 1.

### Behavioral tests

To investigate the presence of anxiety-like behavior in PNA offspring the elevated plus maze and open field tests, two well-established methods to characterize anxiety-like behavior in rodents were used^40^. These tests evaluate the animals’ conflict between their drive to explore novel environments and their avoidance of open spaces. Behavioral tests were performed on 4-month-old female mice. All mice are allowed to rest for at least 45 min in the room where the behavioral tests were done before testing. The elevated plus maze was performed first followed by open field 24 h later. Mice were placed in the central square of the maze and left to freely explore for 5 min. The elevated plus maze consists of an arena elevated 60 cm above the floor comprising four equally spaced arms (10 cm wide, 50 cm long) radiating out from a central square (10 cm). The two opposing closed arms are enclosed by walls (30 cm high), whereas the other two open arms are only guarded by a perimeter border (1 mm high). The maze was placed into a dimly lit room so that light intensity reached 10–15 lx in the closed arms and 50–55 lx in the open arms. The test was recorded with a camera placed above the maze, allowing tracking, and scoring of the mice’s behavior with EthoVision 13 XT (Noldus Information Technology). The entry of the center of the mouse into any of the arms was automatically counted by the EthoVision tracking system. The levels of anxiety were indexed by three variables: the % of time spent in the open arms over the 5 min, the number of entries in the open arms, and the distance moved in the open arms over the total distance moved during the test. The box was cleaned with 70% ethanol followed by water between trials.

The open field tests were conducted in 1 m^2^ plexiglas arenas surrounded by 40 cm high walls (100 × 100 × 40 cm). The arenas were placed in a dimly lit room so that light intensity reached 90 lx in the corners and 240 lx in the center. Mice were placed in a corner of the arenas and left to freely explore for 15 min. The test was recorded with a camera placed above the arenas, allowing tracking, and scoring of the mice’s behavior with EthoVision 13 XT (Noldus Information Technology). A center zone (40 × 40 cm) was defined for scoring in the middle of the arenas. The level of anxiety was indexed by four variables: the latency to enter the center zone, the % of time spent in the center zone, and the distance moved in the center zone. The box was cleaned with 70% ethanol followed by water between trials.

### Tissue collection

One week after the behavioral tests, the mice were fasted for 4 h before blood collection and dissection. Mice were sedated with isoflurane followed by decapitation and dissection.

### Serum and CSF measurements

A small tail blood sample was taken to quantify serum insulin levels by ELISA (Mouse insulin 10–1247-01, Mercodia, Sweden) and glucose was measured with a blood glucose meter (Contour XT, Bayer Health Care, Mishawaka, USA). A larger blood sample for serum analyses was collected right after decapitation. Total and high molecular weight adiponectin in serum was analyzed by ELISA (47-ADPMS-E01, Alpco, Salem, NH, USA) with a sensitivity of 0.032 ng/mL and an intra- and inter-assay CV < 4%. In another cohort of mice, cerebrospinal fluid (CSF) was collected by puncturing the cisterna magna using a glass capillary with a fine tip and gentle suction^41^. Samples with suspected blood contamination were immediately discarded. Total adiponectin was analyzed in serum (diluted 1:50,000) and CSF (diluted 1:100) using an adiponectin ELISA (80569, CrystalChem, Zaandam, The Netherlands) with a sensitivity of 0.008 ng/mL and an intra-assay coefficient variation (CV) < 10%. The absorbance was measured on a plate reader (SpectraMax I3x, Molecular Devices, San Jose, CA, USA).

### Statistical analyses

Statistical analyses were performed with SPSS (version 19.0; SPSS, Chicago, USA). The main effect of PNA, the dam genotype, and the offspring genotype, and any interactions between these three variables, were measured using three-way ANOVA followed by Tukey’s post-hoc test. The variable with a main effect on the studied parameter is included above each graph; PNA, offspring or dam (genotype). To compare adiponectin levels between genotypes, an independent samples t-test was used. Data are expressed as mean ± SEM and *P* < 0.05 was considered significant.

### Supplementary Information

Supplementary Tables.

## Data Availability

The data supporting the findings of this study are available on request with the corresponding author.
