# Supplementary material for: Elevated circulating adiponectin levels do not prevent anxiety-like behavior in a PCOS-like mouse model
Source: Sci Rep. 2024 Jan 4;14:563. doi: 10.1038/s41598-023-50503-8 (PMC10766608; doi:10.1038/s41598-023-50503-8)
Supplement: Supplementary file 1 — Supplementary Tables. [file 41598_2023_50503_MOESM1_ESM.pdf]

Supplemental Table 1. Brown adipose tissue (BAT), liver, and ovary weight in 4-month-old wild-type (wt) and adiponectin transgenic (APNtg) female mice with and without prenatal androgenization (PNA).

	**Wt dam**				**APNtg dam**				
	wt Veh (n= 14)	APNtg Veh (n= 10)	wt PNA (n= 26)	APNtg PNA (n= 16)	wt Veh (n= 18)	APNtg Veh (n= 13)	wt PNA (n= 9)	APNtg PNA (n= 8)	Three-way ANOVA
BAT (mg)	65.6 ±7.4	152.6 ±14.9	59.8 ±4.7	143.0 ±9.6	57.2 ±4.5	131.6 ±11.9	41.6 ±2.1	154.0 ±15.3	Offspring $F_{(1,105)}$ =195, P<0.001
Liver (g)	0.92 ±0.06	0.86 ±0.07	0.83 ±0.03	0.78 ±0.03	0.83 ±0.04	0.80 ±0.04	0.81 ±0.03	0.79 ±0.08	ns
Ovaries (mg)	20.8 ±3.6	14.1 ±0.9	18.3 ±3.1	13.9 ±0.7	21.0 ±2.5	17.8 ±3.4	15.1 ±1.1	14.2 ±1.1	ns

The main effect of PNA, the dams´ genotype and the offsprings´ genotype, as well as any interactions were measured using three-way ANOVA. Data are expressed as mean ± SEM and P < 0.05 was considered significant.

Supplemental Table 2. Open field behavior measurements in 4-month-old wild-type (wt) and adiponectin transgenic (APNtg) female mice with and without prenatal androgenization (PNA).

	**Wt dam**				**APNtg dam**				
	wt Veh (n= 14)	APNtg Veh (n= 10)	wt PNA (n= 24)	APNtg PNA (n= 15)	wt Veh (n= 18)	APNtg Veh (n= 13)	wt PNA (n= 9)	APNtg PNA (n= 8)	Three-way ANOVA
Distance moved (m)	92 ±10	81 ±8	70 ±5	83 ±5	88 ±7	61 ±8	91 ±10	80 ±7	ns
Distance moved in center zone (%)	10.3 ±1.1	6.0 ±1.1	6.8 ±0.5	7.7 ±0.4	8.4 ±0.8	4.8 ±1.6	10.0 ±1.6	9.3 ±2.1	Offspring $F_{(1, 110)}$= 6.11, P=0.015
Latency to enter center zone (s)	25 ±6	89 ±57	25 ±5	36 ±6	36 ±13	50 ±14	34 ±13	44 ±15	ns
Time spent in center zone (%)	6.1 ±1.0	3.8 ±0.7	3.9 ±0.4	4.4 ±0.4	5.6 ±0.6	3.4 ±1.2	6.0 ±0.9	5.3 ±1.7	ns
Entires in center zone (n)	32.1 ±5.0	18.4 ±4.0	19.2 ±2.4	25.6 ±3.1	33.8 ±3.2	27.3 ±6.9	30.4 ±5.6	23.8 ±5.7	ns

The main effect of PNA, the dams´ genotype and the offsprings´ genotype, as well as any interactions were measured using three-way ANOVA. Data are expressed as mean ± SEM and P < 0.05 was considered significant.
